# Ethereal Ethics

**DOI:** 10.1371/journal.pbio.0020189

**Published:** 2004-06-15

**Authors:** Robin Lovell-Badge

## Abstract

In response to the Blackburn and Rowley essay on the President's Council on Bioethics, several thought-provoking opinions on ethical challenges in biomedical research are expressed by prominent stakeholders

It is a great pity when vested interest and dogma dominate what should be a well-informed and rational debate. The essay by Elizabeth Blackburn and Janet Rowley (2004), about the output and the workings of the President's Council on Bioethics, therefore prompted in me a strong reaction of sadness and despair, although I have to admit not one of surprise.

In the United Kingdom, we have had an almost continuous debate since the mid 1980s on topics relating to research on early human embryos. I myself have been involved in some of this debate, especially over the last few years, relating to human embryonic stem cells and nuclear transfer. I will not dwell on the political outcomes of this debate, which are widely known, but I want to stress that it has been one that has been very well informed, with contributions from all sides, including many highly respected moral philosophers and bioethicists. These include notable individuals such as Dame Mary Warnock and bodies such as the Nuffield Bioethics Council, who have been especially valuable because of their independence.

So why are the conclusions reached by bioethicists in the UK, who are generally supportive of research involving human embryos, different from those of the President's Council on Bioethics? The same scientific information is available on both sides of the Atlantic. The rules of logic are the same. So it has to be the way the information is interpreted or filtered. This implies bias or vested interest or the input of dogma that is based on belief rather than rational thought. Some examples of this are discussed in the Blackburn and Rowley essay, and they are very worrying. The scare mongering about preimplantation genetic diagnosis is ridiculous—simple mathematics shows that it is implausible to use this technique to screen the usual number of embryos obtained in one round of in vitro fertilisation for more than two or three genetic traits, while we know that intelligence must rely on many more. I am a great fan of science fiction, but I can recognise it as such. I worry that some members of the President's Council seem unable to do this. Many of these daft ideas were already promoted in a book by Francis Fukuyama (2002), and while they can be a harmless way of promoting debate, they should not be included in documents meant to inform policy makers.

It is certainly very unfortunate if the input of real science in the Council is to be reduced. The scientific issues are complex. For example, we certainly do not know nearly enough about either adult or embryonic stem cells to say which will be the best for therapies, and of course it is possible that both will turn out to be useful for different problems. Both also offer exciting new ways to explore human disease and the influence of genetics and environment without having to rely on human experimentation. But any committee looking into what is ethically acceptable has to be provided with a balanced view of what will be possible in the near future. There is no point in being too speculative, in part because it is also difficult to predict what will be ethically acceptable in the future. If cures come from the use of human embryonic stem cells, then I suspect that there will be widespread acceptance, as happened with heart transplants and with in vitro fertilisation, both of which were initially greeted with horror by many.

It is impossible to have an informed debate without accurate and appropriate information, and there seems little point in having a debate that is not informed. Because of various sensitivities, it seemed to me before the creation of the President's Council on Bioethics that for far too long the issues relating to embryo research had not been considered properly within the United States. The President's Council was therefore an opportunity to redress this situation. But from the evidence I fear it will not succeed. Moreover, it does the general public a disservice to pretend to have a serious committee exploring issues of bioethics when that committee fails to live up to the ideals of impartiality and rationality.
